# Outer membrane protein OMP76 of *Riemerella anatipestifer* contributes to complement evasion and virulence by binding to duck complement factor vitronectin

**DOI:** 10.1080/21505594.2023.2223060

**Published:** 2023-06-16

**Authors:** Sen Li, Yanhua Wang, Rongkun Yang, Xiaotong Zhu, Hongying Bai, Xiaojian Deng, Jiao Bai, Yang Zhang, Yuncai Xiao, Zili Li, Zhengfei Liu, Zutao Zhou

**Affiliations:** a College of Veterinary Medicine, Huazhong Agricultural University, Wuhan, China; b State Key Laboratory of Agricultural Microbiology, Huazhong Agricultural University, Wuhan, China; c Key Laboratory of Preventive Veterinary Medicine in Hubei Province, Huazhong Agricultural University, Wuhan, China; d Hubei Hongshan Laboratory, Huazhong Agricultural University, Wuhan, China

**Keywords:** *Riemerella anatipestifer*, vitronectin, complement evasion, OMP76, virulence

## Abstract

*Riemerella anatipestifer* is an important bacterial pathogen in poultry. Pathogenic bacteria recruit host complement factors to resist the bactericidal effect of serum complement. Vitronectin (Vn) is a complementary regulatory protein that inhibits the formation of the membrane attack complex (MAC). Microbes use outer membrane proteins (OMPs) to hijack Vn for complement evasion. However, the mechanism by which *R. anatipestifer* achieves evasion is unclear. This study aimed to characterise OMPs of *R. anatipestifer* which interact with duck Vn (dVn) during complement evasion. Far-western assays and comparison of wild-type and mutant strains that were treated with dVn and duck serum demonstrated particularly strong binding of OMP76 to dVn. These data were confirmed with *Escherichia coli* strains expressing and not expressing OMP76. Combining tertiary structure analysis and homology modelling, truncated and knocked-out fragments of OMP76 showed that a cluster of critical amino acids in an extracellular loop of OMP76 mediate the interaction with dVn. Moreover, binding of dVn to *R. anatipestifer* inhibited MAC deposition on the bacterial surface thereby enhancing survival in duck serum. Virulence of the mutant strain ΔOMP76 was attenuated significantly relative to the wild-type strain. Furthermore, adhesion and invasion abilities of ΔOMP76 decreased, and histopathological changes showed that ΔOMP76 was less virulent in ducklings. Thus, OMP76 is a key virulence factor of *R. anatipestifer*. The identification of OMP76-mediated evasion of complement by recruitment of dVn contributes significantly to the understanding of the molecular mechanism by which *R. anatipestifer* escapes host innate immunity and provides a new target for the development of subunit vaccines.

## Introduction

*Riemerella anatipestifer* is a short, rod-shaped Gram-negative bacterium that is a member of the *Flavobacteriaceae* [1]. *R. anatipestifer* infection, termed infectious serositis, causes a fibrinous exudate in numerous organs in diverse poultry, especially in ducklings and geese, and is responsible for major losses in the poultry industry worldwide [2,3]. Although no definitive economic cost to the poultry industry is available, the incidence of duck serositis is 5–75% with a high mortality rate, which suggests that the economic loss caused by the disease is extensive. Twenty-one serotypes of *R. anatipestifer* have been identified but the lack of cross-protection between different serotypes leads to poor immunisation effects with existing vaccines [4,5]. Therefore, disease control is dominated by the use of antibiotics [6]. However, increased drug resistance and the emergence of drug-resistant strains may lead to food safety concerns caused by antibiotic residues, as well as a decrease in the array of useful antibiotics for treatment of *R. anatipestifer* infection [7,8]. Serositis is a septicaemic infection that proceeds acutely as *R. anatipestifer* rapidly breaks through host defence barriers after entering the bloodstream in which the bacterium survives and proliferates to cause serious infection. Virulence factors of *R. anatipestifer*, including the capsule-capsular polysaccharide (CPS), lipopolysaccharides, gelatinases, the type IX secretion system (T9SS), outer membrane proteins (OMPs), and other putative virulence factors, which are associated with the viability of this bacterium in blood [9–15]. However, the molecular pathogenic mechanisms of *R. anatipestifer* are still enigmatic.

TonB-dependent receptors (TBdRs) located on the outer membrane of Gram-negative bacteria belong to the family of OMPs which, together with the TonB-ExbB-ExbD complex in the inner membrane, constitute the TonB system [16]. External factors needed by bacteria, including iron, zinc, and copper ions and haem that enter the cytoplasm through the active transport of TBdRs, thereby maintaining normal cellular activity [17,18]. Thirty-one TBdRs have been identified in *R. anatipestifer,* which may play important roles in the interaction between this bacterium and the host [19].

The complement system distinguishes host components from invading microbes by discriminating between evolutionarily conserved pathogen-associated molecular patterns (PAMPs). The complement system is activated *via* the classical pathway (CP), the alternative pathway (AP), and the lectin pathway (LP) [20]. As an important component of the natural immune defence response, the complement system is the first line of protection from invasion by pathogens, but also is a particular focus of immune escape strategies by pathogenic bacteria [21]. Pathogenic bacteria have evolved multiple tactics to resist killing by the host complement system, including the expression of proteases that cleave complement factors, production of complement inhibitory factors, and recruitment of complement regulatory factors [22].

Vitronectin (Vn) is a glycoprotein that is present in serum as a single chain of 75 kD and as truncated variants of 65 kD and 10 kD [23]. Together with complement factor H (FH), C4-binding protein (C4BP), C1-inhibitory factor (C1-INH), and factor I (FI), Vn forms the fluid phase complement regulatory factor [24]. Vn acts as a complement regulator that controls complement activity at the level of formation of the membrane attack complex (MAC). Vn inhibits MAC insertion into the cell membrane by binding to the C5b67 metastable membrane-binding site, and also binds directly to the C9 monomer thereby preventing C9 polymerisation [25]. As the formation of MAC is necessary for killing of Gram-negative bacteria, it is common for pathogenic bacteria to produce surface proteins, including StcE in *Escherichia coli* [26], Opc, OpaA, and Msf in *Neisseria meningitidis* [27,28], and Lpd in *Pseudomonas aeruginosa* [29], that capture Vn to mediate complement escape [30].

Our previous studies showed that an intact T9SS exists in *R. anatipestifer* RA-YM and that T9SS effector proteins and bacterial surface antigens jointly inhibit complement activation, thereby promoting evasion of the bactericidal effect of serum complement [31,32]. These observations indicate that an associated surface antigen in this strain is a complement escape protein. Therefore, here we expressed duck vitronectin (dVn) as a bait protein to capture OMPs of the RA-YM strain. OMP76 as a IreA family TonB-dependent siderophore receptor showed particularly strong binding to dVn. We verified the surface localisation of OMP76 and its ability to bind dVn using fluorescence-activated cell sorting (FACS) and western blot. The interaction between the two proteins inhibited the formation and deposition of MAC on the surface of *R. anatipestifer* and enhanced the serosensitivity of the bacterium to complement-mediated escape, which pinpoints OMP76 as a key escape-associated virulence factor in this important pathogen.

## Materials and methods

### Ethics statement

Animal experiments were performed in accordance with the recommendations for the Care and Use of Laboratory Animals from the Research Ethics Committee, Huazhong Agricultural University, Hubei, China. Procedures in studies involving animals were in accordance with the ethical standards of the institution or practice at which the studies were conducted.

## Bacterial strains, cell lines, and growth conditions

The bacterial strains and plasmids used in the study are listed in Supplementary Table 1. *R. anatipestifer* RA-YM and related strains were grown at 37°C in Tryptic Soy Broth (TSB) (Becton, Dickinson and Company, Franklin Lakes, USA) or Giolitti-Cantoni Broth (GCB) [33]. TSA agar (Becton, Dickinson and Company) and GCB agar were used as solid media. 5% newborn bovine serum was from EVERY GREEN, Zhejiang Tianhang Biotechnology (Huzhou, China).

*E. coli* DH5α was used for plasmid construction, *E. coli* BL21 (DE3) was employed for production of recombinant proteins, and *E. coli* DH10Bac (Weidi Biotechnology, Shanghai, China) was used for packaging and production of recombinant baculovirus. *E. coli* X7213 is autotrophic for diaminopimelic acid (100 μg/mL; Sigma-Aldrich) and was used for conjugative transfer between *E. coli* and *R. anatipestifer*. *E. coli* strains were grown in lysogeny broth (LB) and LB agar at 37°C. The concentrations of antibiotics used were (μg/mL): ampicillin, 50; tetracycline, 10; kanamycin, 50; gentamicin, 7; spectinomycin, 100; and, erythromycin, 4.

Sf9 insect cells for production of recombinant baculovirus and protein purification were grown in Sf−900^TM^ II SFM (Gibco, Grand Island, USA) with shaking incubation (140 rpm) at 27°C.

## Western blotting

OMPs of *R. anatipestifer* RA-YM were extracted and separated based on methods described previously [34]. Far-western blotting was used to identify protein–protein interactions. The extracted OMPs (20 μg per well) were separated by SDS-PAGE and transferred to PVDF membranes (Bio-Rad, Hercules, USA). Membranes were incubated in blocking buffer (5% [w/v] skimmed milk in TBST) for 2 h at room temperature. The dVn protein (0.2 mg), mouse anti Strep II-Tag mAb (1:4000 dilution), and HRP Goat Anti-Mouse IgG (H+L) (1:10000 dilution) (ABclonal) were added sequentially. For pull-down assays, stripped proteins on magnetic beads were detected with mouse anti-Strep II-Tag mAb (1:4000 dilution), mouse anti-His-Tag mAb (1:4000 dilution), and HRP goat anti-mouse IgG (H+L) antibody (1:10000 dilution). Rabbit anti-dVn pAb (1:1000 dilution) and HRP goat anti-rabbit IgG (H+L) (1:10000 dilution) antibody were used to determine the amount of Vn adhered to the bacterial surface for the RA-YM strain. Rabbit anti-dC9 pAb (1:1000 dilution) and HRP goat anti-rabbit IgG (H+L) (1:10000 dilution) antibody were used to determine the C9 monomer and oligomer formation on the bacterial surface.

## Protein pull-down assays

Purifed strep-tagged recombinant dVn protein (120 μg) was incubated with Magrose Strep-Tactin beads (Solarbio, Beijing, China) in 1 mL binding buffer (10 mM Tris-HCl, 150 mM NaCl, 1 mM EDTA, pH 8.0) for 4 h at 4°C. After three washes with 1 mL binding buffer, the beads were incubated with OMPs of *R. anatipestifer* RA-YM (500 μg) overnight at 4°C. The beads were washed three times with binding buffer (1 mL), and bound proteins were eluted with elution buffer (50 μL) (10 mM Tris-HCl, 150 mM NaCl, 1 mM EDTA, 2.5 mM desthiobiotin, pH 8.0) and magnetic separation to collect proteins for LC-MS/MS sequencing (Novegene, Beijing, China). The interaction of His_6_-OMP76, His_6_-OMP76_L5_, His_6_-OMP76_L11_, and His_6_-OMP76_Δ307–315_ with dVn was examined further by His-Tag pull-down assays. Expression and purification of His_6_-OMP76, His_6_-OMP76_L5_, His_6_-OMP76_L11_, and His_6_-OMP76_Δ307–315_ is described in the supplementary material.

## ELISA

The interactions between dVn and His_6_-OMPs and truncated fragments (L1, L2, L4, L6, L7, L8, L9, and L10) of OMP76 whose construction method is described in the supplementary material, were analysed by ELISA. Gradients of the His_6_-tagged proteins (1–7 µg/mL) were coated in 96-well plates and the same gradient concentration of BSA was applied as a control overnight at 4°C. Blocking was performed with blocking buffer (5% [w/v] BSA in PBST) for 2 h at 37°C. The dVn protein (0.5 μg), mouse anti-Strep II-Tag mAb (1:5000 dilution), and HRP goat anti-mouse (H+L) antibody (1:7000 dilution) were added sequentially after 1 h incubation at 37°C. Each well was protected from light and TMB liquid substrate ELISA (100 μL) (GBCBIO Technologies, Guangzhou, China) was added. After sufficient colour development, OD_630_ values were measured with a Multiskan MK3 microplate reader (Thermo, Waltham, USA). Data were plotted for analysis.

## Antibody production

Cloning, expression and purification of His_6_-RecA and His_6_-OMP76, recombinant baculovirus construction and purification of dVn and dC9 are described in the supplementary material. 500 μg of purified His_6_-OMP76, His_6_-RecA, dVn, and dC9 were mixed with complete Freund’s adjuvant, fully emulsified, and immunised to New Zealand white rabbits. Rabbits were boosted with 500 μg of protein on days 14, 28, and 42 after the first immunisation and tested for antibody titres by ELISA. Serum was collected by carotid exsanguination as polyclonal antibodies and stored in aliquots at −80°C.

## Construction of *R. anatipestifer* YMΔOMP76 and YMCΔOMP76 strains

The 5’ and 3’ homology arms of the *omp76* gene were amplified by PCR using the RA-YM strain genome as a template and the spectinomycin resistance gene was amplified using the pIC−333 plasmid as a template. The three fragments were ligated by overlap PCR to obtain the OMP76-LSR product in which the resistance gene is flanked by the homology arms. The *R. anatipestifer* YM**Δ**OMP76 was constructed according to the method described by Liu et al [33]. The method of construction of the complement strain RA-YMCΔOMP76 was described previously [35].

## OMP76 mediated-binding of *R. anatipestifer* RA-YM to dVn

The *E. coli* BL21 (DE3)/pET−16b-OMP76 expression strain was analysed in parallel with *R. anatipestifer* RA-YM strains by flow cytometry for OMP76 expression on the cell surface. The wild-type and RA-YMΔOMP76 strains were grown to log phase and adjusted to concentrations of 1 × 10^9^ CFU/mL. *E. coli* BL21 (DE3)/pET−16b-OMP76 and *E. coli* BL21 (DE3)/pET−16b were inoculated in LB broth with ampicillin for plasmid maintenance and IPTG (1 mM) was added to induce expression when OD_600_ = 0.6–0.8 followed by incubation at 27°C for 8 h at which point the concentration was adjusted to 1 × 10^9^ CFU/mL. Normal duck serum (NDS) was diluted 25 to 500-fold with PBS supplemented with 2% BSA and purified dVn was diluted similarly to a concentration of 1 to 20 μg/mL. 100 μL RA-YM, RA-YMΔOMP76, *E. coli* BL21 (DE3)/pET−16b-OMP76 and *E. coli* BL21 (DE3)/pET−16b were added to each dilution of NDS and dVn protein for 4 h incubation at 4°C. The cells were washed with PBS supplemented with 2% BSA three times before western blotting and FACS analysis to assess differences in the amount of adherent Vn on the bacterial surfaces.

## Fluorescence-activated cell sorting analysis

Rabbit anti-OMP76 pAb (1:50 dilution) and FITC goat anti-rabbit IgG antibody (1:100 dilution) were added to cultures of *R. anatipestifer* RA-YM, *R. anatipestifer* RA-YMΔOMP76, *E. coli* BL21 (DE3)/pET−16b-OMP76, or *E. coli* BL21 (DE3)/pET−16b to detect the OMP76 localisation at the bacterial surface. The dVn protein (2 μg) was added to each sample following with rabbit anti-dVn pAb (1:50 dilution) and FITC goat anti-rabbit IgG antibody (1:100 dilution) for the detection of OMP76 mediated-binding of *R. anatipestifer* RA-YM to dVn by FACS. 10000 individual bacterial cells were detected in each assay using a CytoFLEX-LX instrument (Beckman Coulter, Indianapolis, USA). The positive population was gated at 3% of the background-binding population in the control.

## MAC deposition assay

*R. anatipestifer* RA-YM, *R. anatipestifer* RA-YMΔOMP76, *E. coli* BL21 (DE3)/pET−16b-OMP76, and *E. coli* BL21 (DE3)/pET−16b were prepared using the conditions outlined above. NDS (final concentration 5%) was added to 10^8^ CFU of cell suspension and samples were incubated at 37°C. Aliquots were taken at 10, 20, 30, 40, 50, and 60 min and transferred to ice to terminate the complementation reaction. Loading buffer was added followed by incubation at 95°C for 10 min for western blotting assay.

## Serum survival assay

OMP76-mediated complement escape by *R. anatipestifer* RA-YM was assessed by serum survival assays as outlined in in our previous study [32]. Briefly NDS and heat inactivated NDS (HIS) were diluted with HBSS^++^ (Gibco) to concentrations of 10% and 2% (v/v) and mixed with 10^6^ CFU of RA-YM, RA-YMΔOMP76, RA-YMCΔOMP76, *E. coli* BL21(DE3)/pET−16b-OMP76, or *E. coli* BL21 (DE3)/pET−16b strains. After 30 min incubation at 37°C, samples were diluted serially and three replicates of each dilution were plated for counted.

## Adhesion and invasion assays

Vero cells were digested with trypsin and spread flat in 24-well plates until the cell density reached 2.5 × 10^5^ cells/well and then were incubated with *R. anatipestifer* RA-YM, *R. anatipestifer* RA-YMΔOMP76, or *R. anatipestifer* RA-YMCΔOMP76 (multiplicity of infection = 100) at 37°C in 5% CO_2_ for 1.5 h. The supernatant was aspirated and discarded, and cells were washed in PBS and digested with 0.1% trypsin. Bacteria were serially diluted and plated on TSA agar to assess the numbers. After washing, DMEM containing gentamicin (100 μg/mL) was added with incubation at 37°C in 5% CO_2_ for 1 h to kill extracellular bacteria and to determine the number of invading bacteria.

## Pathogenicity analysis of *R. anatipestifer* RA-YM strain, *R. anatipestifer* RA-YMΔOMP76 and *R. anatipestifer* RA-YMCΔOMP76 in ducklings

*R. anatipestifer* RA-YM and *R. anatipestifer* RA-YMΔOMP76 were grown to log phase (OD_600_ = 0.6–0.8) in TSB medium, harvested, washed three times with sterile PBS, resuspended, and diluted to 5 × 10^9^, 5 × 10^8^, 5 × 10^7^, 5 × 10^6^, and 5 × 10^5^ CFU/mL. Determination of bacterial virulence was performed as previously described [34]. Tissue lesions were was scored as: 0 (Normal), no obvious lesions were found in all tissues; 1 (Mild), the capsules of the heart, liver, spleen and brain were thickened, the area with cellulous exudation and fatty degeneration of cells was <25%, the area around blood vessels was oedematous, the area with inflammatory cell infiltration was <25%, and the area with neurolysis was <25%; 2 (Moderate), the area of lesions in each tissue as >25% and <50%; 3 (Severe), the area of lesions in each tissue was >50% and <75%; and, 4 (Very Severe), the area of lesions in each tissue was >75% and the total score was 16.

## Statistical analyses

Statistical analysis was performed using GraphPad Prism version 8.0 (GraphPad, LA Jolla, USA). A T-test was used for the analysis of geometric mean, one-way ANOVA was used for adhesion and invasion assays, and two-way ANOVA was used for other assays. The significance level for all analyses was set as *P ≤0.05, **P ≤0.01 and ***P ≤0.001.

## Results

### *Expressed duck vitronectin interacts with several outer membrane proteins of the* R. anatipestifer *RA-YM strain*

**Figure 1.**
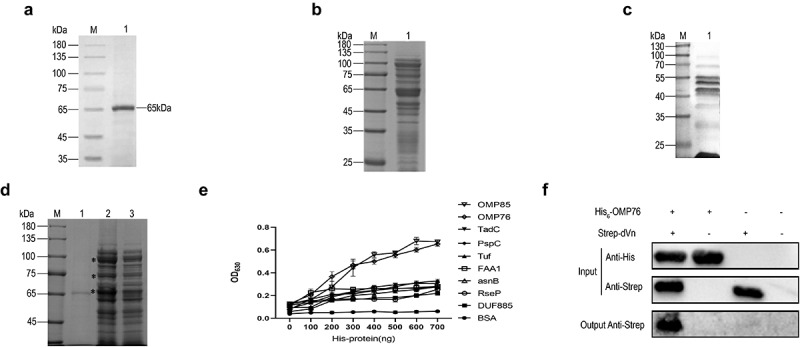
Screening for *R. anatipestifer* OMPs that interact with dVn. (a) SDS-PAGE detection of purified dVn. Lanes: M, molecular weight marker; 1, purified dVn. (b) OMPs of *R. anatipestifer* RA-YM were detected by SDS-PAGE and mainly were in the range of 20–120 kD. (c) Identification (by far-western blot) of OMPs of *R. anatipestifer* RA-YM that interact with dVn. (d) Pull-down with dVn of OMPs of the RA-YM strain by SDS-PAGE. Lanes: 1, purified dVn; 2, dVn pull down of OMPs; 3, negative control. (e) ELISA validation of His-tagged OMPs that interact with dVn. (f) Western blot validation of His_6_-OMP76 pull down using Strep-dVn. Three independent experiments were conducted and a representative experiment is shown here. The nature of data points belongs to biological repetition.

The dVn gene sequence was amplified by PCR (1356 bp) and the expression of the Strep-tagged dVn protein from the recombinant baculovirus was detected by western blot and immunofluorescence using anti-Strep antibody in Sf9 insect cells (Suplementary Figure S1). Strep-dVn protein was expressed abundantly under toxigenic conditions (multiplicity of infection = 2). The purity of the Strep-dVn protein was assessed by SDS-PAGE (Figure 1a). Cloning, expression and purification of Strep-dC9 protein are shown in Supplementary Figure S2.

OMPs were extracted from *R. anatipestifer* RA-YM at a concentration of 9.8 mg/mL and were detected by SDS-PAGE (Figure 1b). Far-western blotting and Strep pull-down assays demonstrated that numerous proteins in the extract associated with dVn (Figure 1c,d). The interacting OMPs from these pull-down assays were subjected to mass spectrometry sequencing, which revealed that nine proteins that bind to dVn were selected with high confidence: OMP85 (KYF39_05195), OMP76 (KYF39_07405), TadC (KYF39_02070), FAA1 (KYF39_03210), asnB (KYF39_03740), DUF885 (KYF39_05185), RseP (KYF39_06290), Tuf (KYF39_07635), and PspC (KYF39_08600). Purification of His-tagged versions of these nine candidate proteins is shown in Supplementary Figure S3. The interactions between these proteins and dVn were verified by ELISA experiments, which indicated that OMP76 and OMP85 interacted most avidly with dVn (Figure 1e). In this study, we first characterised OMP76 and its interaction with dVn which was validated by His pull-down assays (Figure 1f).

## Amino acids 307–315 mediate the interaction between OMP76 and duck vitronectin

**Figure 2.**
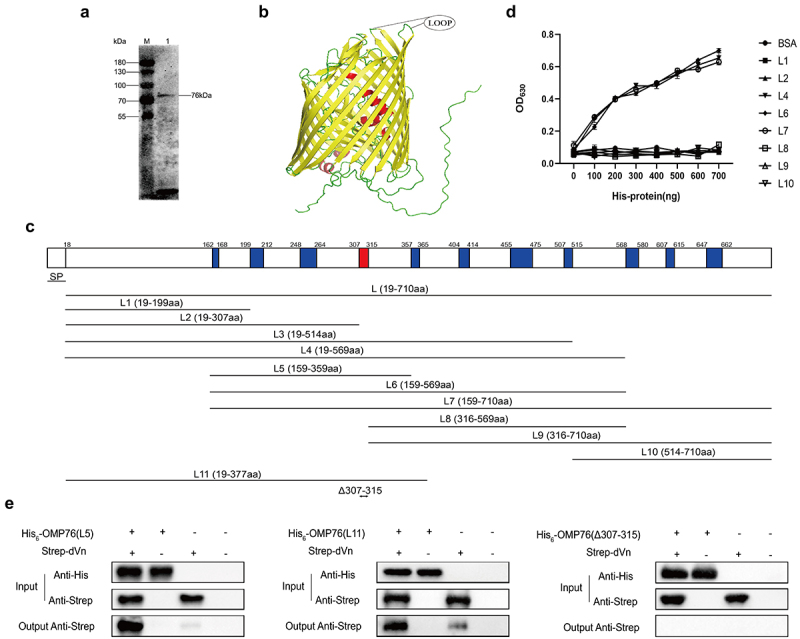
Expression of truncated OMP76 and screening for the OMP76 domain that interacts with dVn. (a) Detection of the immunogenicity and reactogenicity of OMP76 with R. anatipestifer RA-YM whole bacteria immune serum by Western blotting. The antibody used was a duck anti-R. anatipestifer RA-YM strain whole protein immune serum prepared in our laboratory, and the secondary antibody was HRP*PCAB rabbit anti-duck IgY (IgG)(H+L) (Cellwaylab, Luoyang, China). (b) the tertiary structure of OMP76 predicted by AlphaFold. Red, helix; yellow, β-strand; green, loop. (c) the position of truncated fragments derived from full-length OMP76. (d) ELISA analysis of the interaction of truncated segments of OMP76 with dVn. (e) Pull-down assay of the interaction of truncated segments L5 and L11, and knockdown fragment OMP76_Δ307–315_ with dVn by Western blotting. ELISA and pull-down assays were repeated three times and representative experiments are shown here. The nature of data points belongs to biological repetition.

OMP76 is composed of 710 amino acids with a putative signal peptide located at amino acids 1–18 using SignalP−5.0 analysis. Thus, the mature protein is predicted to contain 692 residues with a molecular weight of 76 kD. OMP76 reacts with *R. anatipestifer* RA-YM whole bacterial immune serum, indicating that the protein possesses effective immunogenicity and reactogenicity (Figure 2a). The tertiary structure of OMP76 was found in AlphaFold (http://www.alphafold.com; gene ID RA0C_1745). OMP76 is predicted to be a barrel-shaped transmembrane protein consisting of 22 reverse parallel β-folds with 11 extracellular loops. The protein is similar to TonB-dependent receptors and shares 22.07% homology with extraintestinal pathogenic *E. coli* TonB-dependent receptor iron regulatory protein IreA. Therefore, based on the tertiary structure (Figure 2b) and secondary structure predictions (Supplementary Figure S4), OMP76 was divided into 10 truncated overlapping segments: L1 (amino acids 19–199), L2 (amino acids 19–307), L3 (amino acids 19–514), L4 (amino acids 19–569), L5 (amino acids 159–359), L6 (amino acids 159–569), L7 (amino acids 159–710), L8 (amino acids 316–569), L9 (amino acids 316–710), and L10 (amino acids 514–710). Apart from L3 and L5, these fragments were expressed and purified as His-tagged peptides from the pET−22b vector (Figure 2c and Supplementary Figure S5). The ability of the eight purified segments of OMP76 to interact with dVn was examined by ELISA which showed that L4, L6, and L7 independently bound to dVn, but that the remaining five fragments did not (Figure 2d). Based on this analysis, including the observation that L2, which comprises amino acids 19–307 does not bind to dVn, we tentatively identified that amino acids 307–315 mediate the interaction of the OMP76 protein with dVn. Next, we produced L11 (amino acids 19–377) that includes the proposed binding site for dVn (Figure 2c), and also used pET−32a to express the L5 fragment that was not produced from pET−22b. A derivative, OMP76_Δ307–315_, with a deletion of amino acids 307–315 also was generated (Supplementary Figure S6). Using L11, L5 and OMP76_Δ307–315_ as His-tagged bait proteins, we examined binding to dVn in pull-down assays, which showed that L11 and L5 interacted with dVn, but that the deletion in OMP76_Δ307–315_ abolished this interaction (Figure 2e). These data confirm that amino acids 307–315 are key residues to the interaction of OMP76 with dVn.

## OMP76 mediates binding of *R. anatipestifer* RA-YM cells to vitronectin

**Figure 3.**
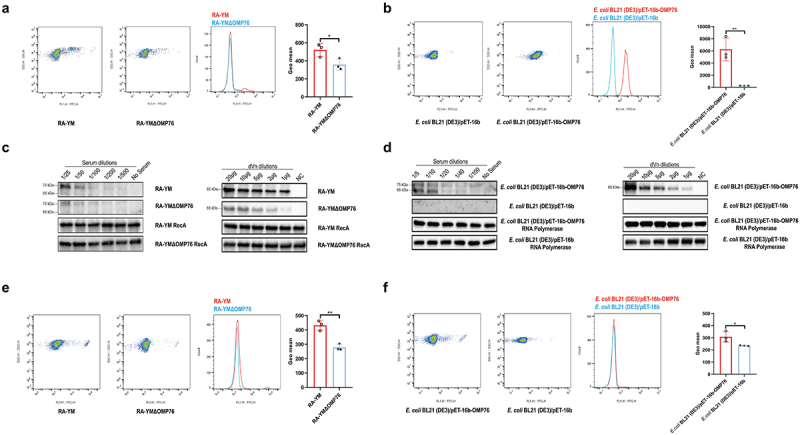
Validation of the surface localisation of OMP76 and the ability of OMP76 to mediate bacterial binding of dVn. (a) FACS analysis of *R. anatipestifer* RA-YM and R. anatipestifer RA-YMΔOMP76 binding to OMP76 pAb. (b) FACS analysis of E. coli BL21(DE3)/BL21(DE3)/Pet−16b−16b-OMP76 and E. coli BL21(DE3)/pET−16b binding to OMP76 pAb. (c) Western blot assay of binding of the RA-YM and RA-YMΔOMP76 strains to dVn in normal duck serum and to purified dVn protein. (d) Western blot assay of *E. coli* BL21(DE3)/BL21(DE3)/Pet−16b−16b-OMP76 and E. coli BL21(DE3)/pET−16b binding to dVn in normal duck serum and to purified dVn protein. (e) FACS analysis of binding of the RA-YM and RA-YMΔOMP76 strains to purified dVn protein. (f) FACS analysis of binding of E. coli BL21(DE3)/BL21(DE3)/Pet−16b−16b-OMP76 and E. coli BL21(DE3)/pET−16b to purified dVn protein. A representative image of three independent experiments is shown. The nature of data points belongs to biological repetition.

In view of the preceding experiments, we used FACS to examine the ability of *R. anatipestifer* RA-YM and *R. anatipestifer* RA-YMΔOMP76 that is deleted of the gene for OMP76 to bind anti-OMP76 antibody (Figure 3a). 809, 310, 8099, and 10 cells of *R. anatipestifer* RA-YM, *R. anatipestifer* RA-YMΔOMP76, *E. coli* BL21(DE3)/pET−16b-OMP76, and *E. coli* BL21(DE3)/pET−16b, respectively, bound the OMP76 polyclonal antibody among every 10,000 cells. The mean fluorescence values of the wild-type and deletion strains were significantly different. Moreover, *E. coli* BL21(DE3)/pET−16b-OMP76 that expressed the OMP76 protein bound the antibody more avidly than *E. coli* BL21(DE3)/pET−16b that does not produce OMP76 (Figure 3b). These data demonstrate the surface localisation of OMP76 in both *R. anatipestifer* and in *E. coli* that produces the protein ectopically.

We next tested the ability of the wild-type RA-YM strain, the RA-YMΔOMP76 deletion strain, and *E. coli* strains that do and do not express OMP76 to bind dVn in NDS to which purified dVn protein was added. Western blot results showed, first, that the capacity of these strains to bind dVn decreased gradually with increasing dilution of NDS and purified dVn protein. Second, the binding of the RA-YMΔOMP76 strain and of *E. coli* BL21(DE3)/pET−16b to dVn was significantly lower than binding by the RA-YM strain or *E. coli* BL21(DE3)/pET−16b-OMP76 (Figure 3c,d). Binding to dVn was verified by FACS analysis of equal concentrations of the four strains that were incubated with 2 μg of dVn protein followed by rabbit anti-Vn polyclonal antibody and FITC goat anti-rabbit IgG antibody. The mean fluorescence values of wild-type *R. anatipestifer* and of *E. coli* expressing OMP76 were significantly higher than the RA-YMΔOMP76 strain and *E. coli* without OMP76, respectively, which confirms that OMP76 mediates bacterial binding to dVn (Figure 3e,f).

## Binding of vitronectin by OMP76 inhibits the deposition of the membrane attack complex on the bacterial cell surface

**Figure 4.**
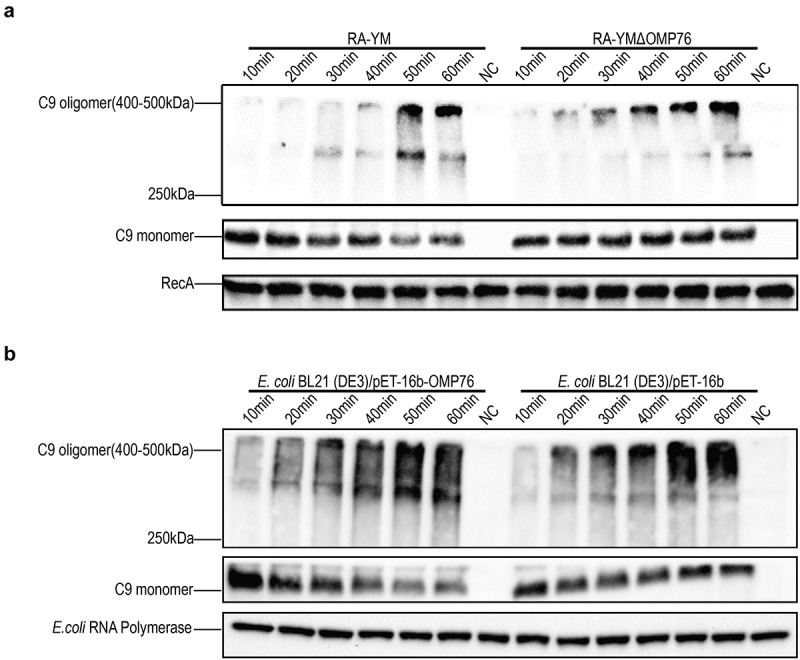
Western blot detection of C9 multimers deposited on the bacterial cell surface. (a) Western blotting assay of C9 deposition on *R. anatipestifer* RA-YM and R. anatipestifer RA-YMΔOMP76 when incubated in 5% NDS for the indicated time periods. (b) Western blotting assay of C9 deposition on E. coli BL21(DE3)/BL21(DE3)/Pet−16b−16b-OMP76 and E. coli BL21(DE3)/pET−16b when incubated in 5% NDS for the indicated time periods. (a) and (b) were repeated three times and representative images of these independent experiments are shown.

The Vn protein binds directly to C5b67 or C9 monomers of the complement system and thereby inhibits the formation of MAC. Therefore, we used the production of serum complementation factor C9 multimers to assess the deposition of MAC on the surface of *R. anatipestifer* and *E. coli*. Western blotting was performed to detect differences in MAC formation on the cell surfaces. MAC deposition with the RA-YMΔOMP76 strain and *E. coli* BL21(DE3)/pET−16b was evident at 30 min, whereas MAC formation on the surfaces of wild-type *R. anatipestifer* and *E. coli* BL21(DE3)/pET−16b-OMP76 was apparent only after 50 min of incubation (Figure 4a,b). Thus, the binding of dVn to *R. anatipestifer* and *E. coli* that produce OMP76 significantly inhibits MAC deposition on the cell surfaces.

## Serum survival of *R. anatipestifer* and *E. coli* correlates with OMP76 production

**Figure 5.**
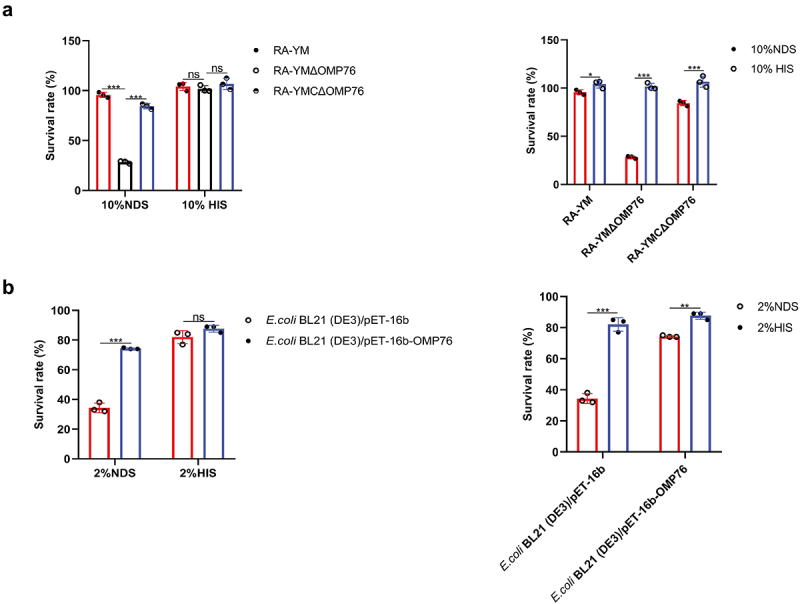
Serum survival assays. (a) Serum survival of R. anatipestifer RA-YM, R. anatipestifer RA-YMΔOMP76, and R. anatipestifer RA-YMCΔOMP76 in 10% NDS. (b) Serum survival of *E. coli* BL21(DE3)/BL21(DE3)/Pet−16b−16b-OMP76 and *E. coli* BL21(DE3)/pET−16b in 2% NDS. Panels (a) and (b) also show the survival of the RA-YM, RA-YMΔOMP76, and RA-YMCΔOMP76 strains and *E. coli* BL21(DE3)/BL21(DE3)/Pet−16b−16b-OMP76 and *E. coli* BL21(DE3)/pET−16b in HIS at the same serum dilution concentrations thereby assessing complement activity in serum. The data were analysed by two-way ANOVA and error bars represent standard deviations.

The preceding results suggested that bacterial binding to dVn exerted an inhibitory effect on complement activation. This observation was tested further by serum survival assays. The serum viabilities of *R. anatipestifer* RA-YM, RA-YMΔOMP76 and RA-YMCΔOMP76 in 10% NDS were approximately 95%, 30% and 85%, respectively, with significant differences between the three (*P* ≤0.001). No significant differences between the strains in 10% HIS were evident (Figure 5a). Similarly, serum viabilities of *E. coli* strains that produced OMP76 or did not express the protein in 2% NDS were approximately 80% and 40%, respectively (*P* ≤0.01), whereas no significant difference was evident with 2% HIS (Figure 5b). Thus, production of the OMP76 protein correlated significantly with the serum survival of both *R. anatipestifer* and *E. coli*. We also tested the activity of complement in NDS: survival of the strains in HIS in which complement is inactivated was higher than in NDS, which indicates that complement may play a role in bacterial persistence in serum.

*P≤0.05; **P≤0.01; ***P≤0.001. The nature of data points belongs to biological repetition.

## Deletion of *omp76* leads to attenuated virulence of *R. anatipestifer* RA-YM

Disruption of the *omp76* gene in *R. anatipestifer* was achieved by homologous recombination using a linear PCR product in which the 5’ and 3’ ends of the gene were separated by a spectinomycin resistance gene. This product was introduced into *R. anatipestifer* RA-YM by natural transformation and isolates that grew on TSA plates containing spectinomycin were identified. The *omp76* gene potentially was disrupted in these candidates. Accordingly, the spectinomycin resistance gene and a control 16S RNA fragment were amplified from these candidates, but the *omp76* gene was not detected (Supplementary Figure S7). In contrast, *omp76* was amplified from the wild-type strain. These results indicated the construction of *R. anatipestifer* RA-YMΔOMP76 in which the *omp76* gene was disrupted. The wild-type and deletion strains were compared in pathogenicity assays in ducklings as outlined in Materials and Methods. LD_50_ measurements after 7 days of infection showed that the parental strain was present at 3.16 × 10^6^ CFU/ml whereas the RA-YMΔOMP76 strain and the RA-YMCΔOMP76 strain were present at 5.77 × 10^8^ CFU/ml and 1.15 × 10^7^ CFU/ml, respectively. Thus, the deletion of the *omp76* gene decreased the CFU/ml approximately 183-fold compared with the wild-type strain. Moreover, fewer deaths occurred in ducklings infected with the deletion strain than the wild-type strain (Figure 7a). These data reveal that *omp76* is an important pathogenicity factor in *R. anatipestifer* during infection.

### R. anatipestifer *RA-YM deleted of* omp76 *is less virulent than the wild-type strain in Vero cells*

**Figure 6.**
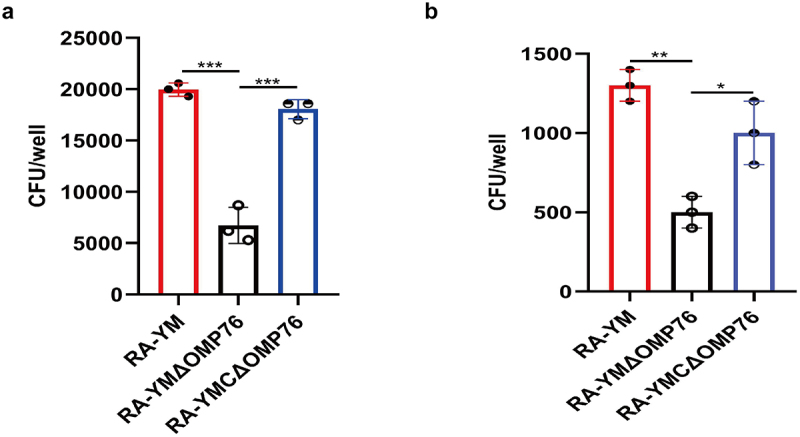
Adhesion and invasion assays of R. anatipestifer RA-YM, R. anatipestifer RA-YMΔOMP76, and R. anatipestifer RA-YMCΔOMP76. (a) Adhesion assays of the RA-YM, RA-YMΔOMP76, and RA-YMCΔOMP76 strains to Vero cells. (b) Invasion assay of the RA-YM, RA-YMΔOMP76, and RA-YMCΔOMP76 strains to Vero cells. All data are mean values of three independent experiments that were analysed by one-way ANOVA. Error bars represent the standard deviations.

To determine whether the OMP76 protein is involved in the ability of *R. anatipestifer* to adhere and invade cells, the wild-type RA-YM, RA-YMΔOMP76, and RA-YMCΔOMP76 strains were used to conduct adhesion and invasion tests with Vero cells. The adhesion and invasion of the RA-YMΔOMP76 strain was significantly weaker than the wild-type strain, whereas adhesion and invasion of Vero cells by RA-YMCΔOMP76 was enhanced compared with the deletion strain (Figure 6a,b). Thus, the *omp76* locus is key to adhesion and invasion by *R. anatipestifer*.

*P ≤ 0.05; **P ≤ 0.01; ***P ≤ 0.001. The nature of data points belongs to biological repetition.

## Knock-out of *omp76* in R. anatipestifer RA-YM reduces tissue bacterial loads and mitigates pathological lesions

**Figure 7.**
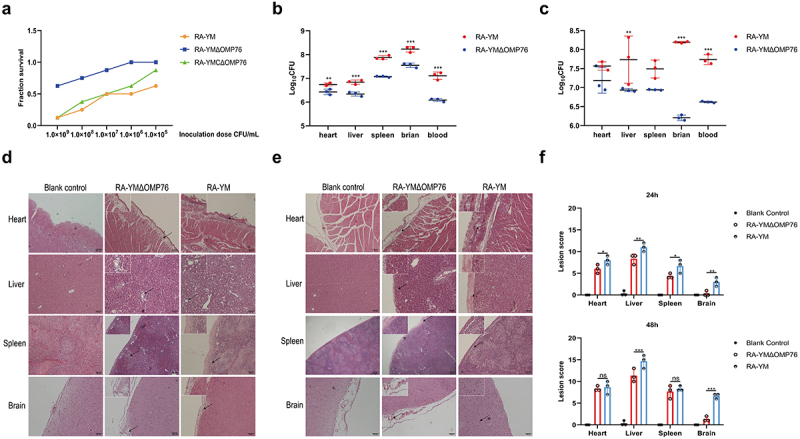
Determination of blood and tissue load of ducklings infected with R. anatipestifer RA-YM or R. anatipestifer RA-YMΔOMP76. (a) Mortality of ducklings infected with R. anatipestifer RA-YM, R. anatipestifer RA-YMΔOMP76, and R. anatipestifer RA-YMCΔOMP76. (b) Bacterial load in the heart, liver, spleen, and brain of Cherry Valley ducks infected with the RA-YM or RA-YMΔOMP76 strains for 24 h. (c) Bacterial load in the heart, liver, spleen, and brain of Cherry Valley ducks infected with the RA-YM or RA-YMΔOMP76 strains for 48 h. Two-way ANOVA was used to assess differences in bacterial numbers in different tissues. *P≤0.05; **P≤0.01; ***P≤0.001. (d) Histopathological analysis of ducklings infected with the RA-YM or RA-YMΔOMP76 strains for 24 h. (e) Histopathological analysis of ducklings infected with the RA-YM or RA-YMΔOMP76 strains for 48 h. (f) Lesion score of the heart, liver, spleen, and brain of Cherry Valley ducks infected with the RA-YM or RA-YMΔOMP76 strains for 24h and 48 h. Two-way ANOVA was used to assess differences in lesion score in different tissues. *P≤0.05; **P≤0.01; ***P≤0.001. The nature of data points belongs to biological repetition.

The preceding data indicated that OMP76 was a crucial virulence factor in *R. anatipestifer,* which was examined further by assessing the loads in infected ducklings of strains expressing and not expressing *omp76*. Loads of the RA-YMΔOMP76 strain that lacked *omp76* in blood, spleen, liver, and brain tissues were lower than those of the wild-type strain after both 24 h (*P* ≤0.01) and 48 h (*P* ≤0.01) of infection (Figure 7b,c). Moreover, examination of pathological tissue sections revealed that heart, liver, spleen, and brain lesions after infection with the deletion strain were fewer than that with the wild strain (Figure 7d,f). These observations confirm the vital role of OMP76 during *R. anatipestifer* infection.

## Discussion

*R. anatipestifer* is one of the most important bacterial pathogens that impact the poultry industry [36]. The bacterium deploys several strategies for evasion of killing by the host immune system. The complement system, as an important component of innate immunity, plays an important role in early immunity against *R. anatipestifer* infection [37]. Pathogens have evolved multiple mechanisms to escape killing by the complement system, including the use of surface complement evasion-related proteins to recruit complement regulatory factors and inhibition of activation of complement components on the surface of the organism [38,39]. However, the specific tactics used by *R. anatipestifer* to circumvent the bactericidal effect of complement are uncertain.

OMPs of Gram-negative bacteria are known virulence factors and pathogenic species use these surface proteins to recruit complement regulators, including Vn, C4 binding protein, and complement factor H, for immune escape [40–42]. The Vn protein is an inhibitor of the MAC system *via* the lytic pathway, thereby hindering the terminal pathway of complement activation [43]. Moreover, Vn also plays important roles in mediating bacterial adhesion, cell signalling, and complement escape in pathogenic bacteria [44–46]. In this study, we report that *R. anatipestifer* RA-YM recruits Vn *via* OMP76, thereby inhibiting the activation of complement components on the surface of the bacterium, which promotes immune evasion by the pathogen.

The Strep tag II tagged dVn produced by recombinant expression and purification was 65 kD, which is slightly larger than expected. Human Vn has three glycosylation sites, which may indicate that dVn also is glycosylated and conformationally similar to the native protein [47]. The OMPs here were found by LC-MS/MS sequencing to be admixed with certain bacterial secretory and cytosolic proteins. The data were filtered for high protein false discovery rate confidence, protein size between 21 and 151 kD, number of unique peptides ≥ 1, matching rate of map > 10, coverage rate of peptide ≥ 5, and peptide coverage using relevant annotations from the KEGG, COG and interProScan databases. This sifting produced nine OMPs most likely to interact with dVn (Supplementary Materials Table S3) which were selected, expressed, and purified as His_6_-tagged proteins (Tuf, OMP85, FAA1, DUF885, RseP, AsnB, TadC, OMP76, and PspC). To further screen for interaction with dVn, we validated the binding of OMP76 to dVn by ELISA and His pull-down assays (Figure 1).

Determining the specific region of OMP76 that interacts with dVn is important in dissecting the mechanism by which the proteins are associated. Therefore, we divided OMP76 into 11 overlapping fragments and, using ELISA and His pull-down assays, we tentatively determined that the interaction with dVn involved the region between amino acids 307–315 of OMP76. Deletion of this region disrupted the interaction with dVn (Figure 2) which suggests that the extracellular fourth loop is a critical domain for capture of dVn by OMP76. OMP76 reacted specifically with immune serum of whole proteins of *R. anatipestifer* RA-YM, indicating the excellent immunogenicity and reactogenicity of the protein. Moreover, OMP76 is highly conserved among diverse *R. anatipestifer* serotypes, ranging from 95.21% to 100%, which suggests that the protein may be a promising candidate antigen for subunit vaccines that promote cross protection among different serotypes. The TbdR1 protein of *R. anatipestifer* Th4 produces immune protection against type 1, type 2, and type 10 serotypes of *R. anatipestifer* [48] and OMP76 has potential to broaden the range of vaccines that are available. Determination of the specific domain of OMP76 that interacts with dVn will allow resolution of the masking of Vn binding by antibodies raised after immunisation with OMP76, as well as the possible enhancement of complement bactericidal activity which has been previously validated in *Haemophilus influenzae* [49]. The specific mode of recognition will be determined after fully elucidating the interaction domains of OMP76 and dVn.

FACS assays verified that OMP76 mediates the recruitment of dVn by *Riemerella anatipestifer* RA-YM. The relatively low-binding rate by *R. anatipestifer* RA-YM in these assays may reflect that OMP76 in different stages of the cell cycle inserts with different efficacies in the bacterial outer membrane in reactions catalysed by the BamA cluster, similarly to *E. coli* FepA. or that not all *R. anatipestifer* RA-YM cells possess OMP76 antibody-binding sites [50]. As *R. anatipestifer* RA-YM encodes 31 TbdR homologs, an outer membrane receptor similar in structure to OMP76 May bind non-specifically to the OMP76 antibody and promoted a positive FACS signal among 3% of the RA-YMΔOMP76 cells. In conjunction with the tertiary structure conformational analysis of OMP76, we demonstrated the surface localisation of OMP76. Similar to *Rickettsia*, *Yersinia pestis* and *H. influenzae* [51–53], *R. anatipestifer* RA-YM recruited both forms of Vn in serum (75 kD and 65 kD), whereas only one interacting species was evident when purified dVn protein was used as a bait (Figure 3). The recruitment of the RA-YM strain and *E. coli* expressing OMP76 to purified dVn protein was validated further by FACS. The lower positivity detected in this assay may indicate that the concentration of dVn protein used was insufficient to allow all cells to bind the protein, which produced a weaker fluorescence signal.

The recruitment of Vn to the surface of *R. anatipestifer* RA-YM and *E. coli* that expresses OMP76 strain, causing Vn binding to C5b67 and C9 monomers deposited on the bacterial surfaces, resulted in a lag in the formation of the MAC complex on the surfaces of these strains (Figure 4). The serum survival of the strains was boosted as a consequence (Figure 5). In contrast, depletion of OMP76 in the RA-YMΔOMP76 strain and in *E. coli* that did not produce OMP76 significantly reduced the survival rate in NDS which further indicates that OMP76 is a critical protein that assists *R. anatipestifer* escape of complement bactericidal action. The major surface protein A2 (UspA2) of *Moraxella*, *P. aeruginosa* complement regulatory acquisition surface protein (CRASP−2), and *H. influenzae* surface fibre (Hsf) also capture Vn to bacterial surfaces and mediate serum resistance [54–56].

OMPs of Gram-negative bacteria, including OmpA of *R. anatipestifer* and *Salmonella typhimurium* and *E. coli* SurA, commonly are associated closely with virulence [57–59]. *R. anatipestifer* RA-YMΔOMP76, which is deleted of *omp76* showed a 183-fold decrease in virulence and decreased loads in blood, liver, spleen, and brain in pathogenicity tests in Cherry Valley ducks (Figure 7). Moreover, the adhesion and invasion abilities of this strain to Vero cells also decreased significantly (Figure 6) which further indicates that OPM76 is a virulence factor in *R. anatipestifer*.

Here, we identified OMP76 as a virulence factor in *R. anatipestifer* and a complement escape-associated OMP that plays an important role in pathogenesis by this bacterium. Antibiotics (gentamicin, neomycin, and spectinomycin) currently are the principal strategy for preventing and treating *R. anatipestifer* infections but, combined with the misuse of antibiotics, this approach has caused the emergence of numerous variants that are resistant to diverse types of antibiotics [60], for example, ribosomal RNA methyltransferase-mediated resistance to macrolides and resistance to tetracyclines mediated by *tet(X)* genes [61,62]. Therefore, novel strategies based on immune and pathogenic mechanisms are required to prevent and treat *R. anatipestifer* infections. Vaccination to block *R. anatipestifer* infection is both a reliable and cost-effective method and a viable alternative to antibiotic use [63]. However, inactivated vaccines have a low antigenic load and require booster immunisation, and attenuated vaccines are effective but may recombine with wild-type strains and result in the re-emergence of virulent strains. Moreover, neither inactivated nor attenuated vaccines will solve effectively the problem of lack of cross-protection for different *R. anatipestifer* serotypes as these vaccines potentially only provide immune protection for closely-related serotypes. Therefore, genetically engineered subunit vaccines will become an important tool to solve the problem of cross-protection against *R. anatipestifer*. One or more proteins with effective immunogenicity and highly conserved properties may provide this cross-protection for multiple *R. anatipestifer* serotypes. OMP76 characterised in this study is to complement the escape protein. Most complement escape-associated surface proteins have good immunogenicity, are highly conserved among bacterial species, and possess suitable physicochemical properties that make them promising vaccine targets. Screening strategies using complement escape proteins as cross-protective vaccine antigens have been applied successfully in the development of *Neisseria meningitidis* and *Bordetella pertussis* vaccines [64–66]. Therefore, OMP76 May provide a new target for the development of novel genetically engineered subunit vaccines that provide broad protection against *R. anatipestifer* infection.

## Supplementary Material

Supplemental Material

## Data Availability

The original contributions presented in the study are included in the article and Supplementary Materials. Further inquiries can be directed to the corresponding author. The mass spectrometry raw files have been deposited to ProteomeXchange (https://proteomecentral.proteomexchange.org/cgi/GetDataset?ID=PXD039753) with the accession number PXD039753.
