# Supplementary material for: Outer membrane protein OMP76 of Riemerella anatipestifer contributes to complement evasion and virulence by binding to duck complement factor vitronectin
Source: Virulence. 2023 Jun 16;14(1):2223060. doi: 10.1080/21505594.2023.2223060 (PMC10281475; doi:10.1080/21505594.2023.2223060)
Supplement: Supplemental Material [file KVIR_A_2223060_SM8266.docx]

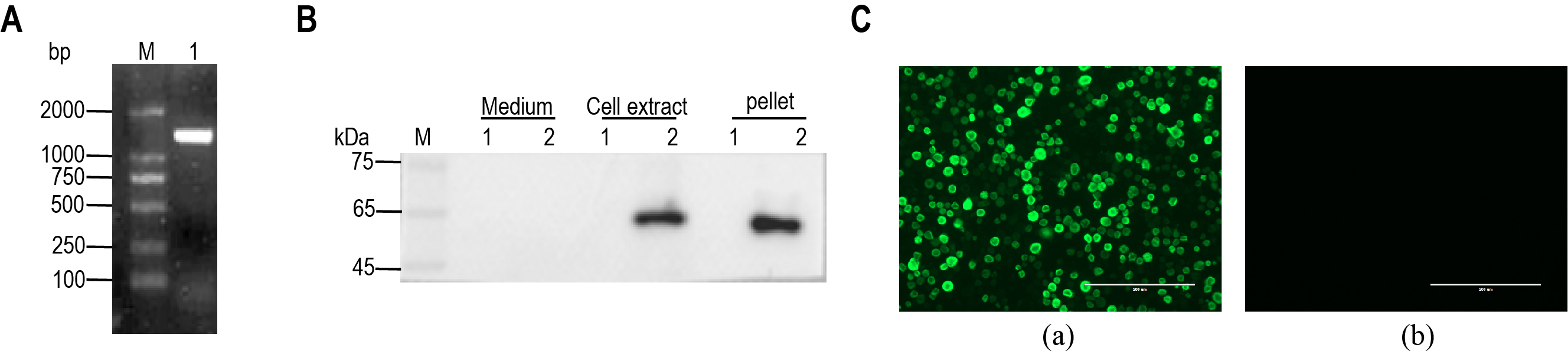


**Supplementary Figure 1.** Cloning and expression of dVn. (A) PCR amplification of Vn. Lanes: M, molecular weight marker; 1, Vn gene product. (B) Western blot of dVn expression in Sf9 cells using anti-Strep antibodies. Lanes: M, molecular weight marker; 1, negative control; 2, identification strep-dVn by Western Blot. (C) Immunofluorescence detection of dVn expression in Sf9 cells using anti-Strep antibodies. (a) Infected Sf9 cell by Bacmid-Vn; (b) Negative control.


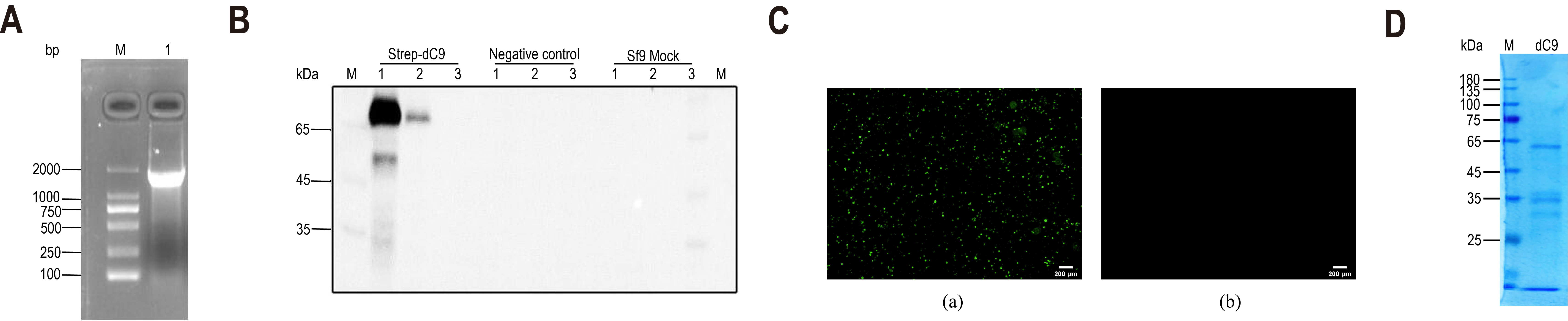


**Supplementary Figure 2.** Cloning and expression of dC9. (A) PCR amplification of dC9.

Lanes: M, molecular weight marker; 1: dC9 gene product. (B) Western blot detection of dC9 expression in Sf9 cells. Lanes:1, medium; 2, cell extract; 3, pellet. (C) Immunofluorescence detection of dC9 expression in Sf9 cells using anti-Strep antibodies. (a) Infected Sf9 cell by Bacmid-C9; (b) Negative control. (D) SDS-PAGE detection of purified dC9.


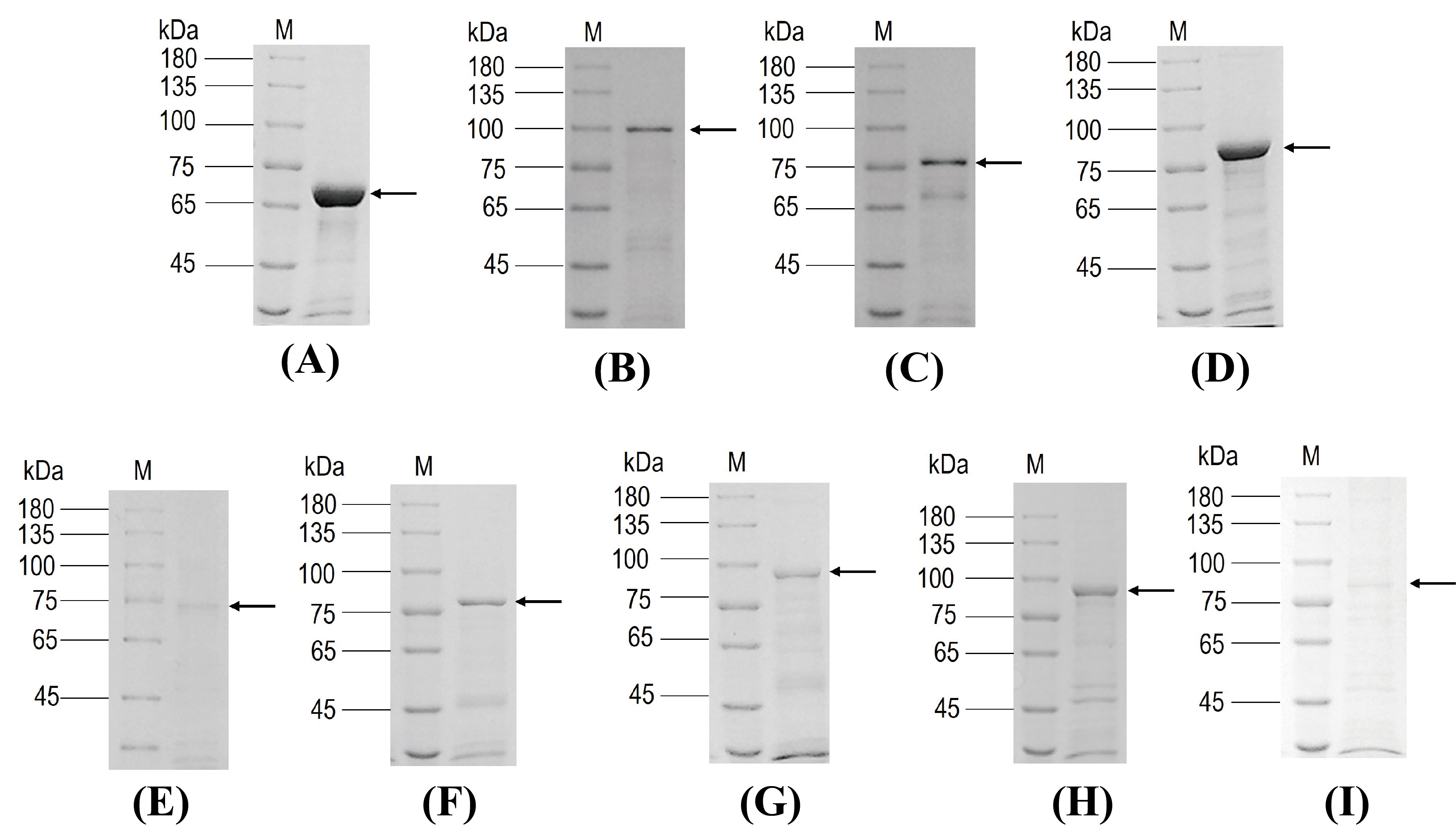


**Supplementary Figure 3.** SDS-PAGE of the nine purified OMPs that interact with dVn. (A) Tuf, (B) OMP85, (C) FAA1. (D) DUF885. (E) RseP. (F) asnB. (G) TadC. (H) OMP76. (I) PspC. M, molecular weight marker.


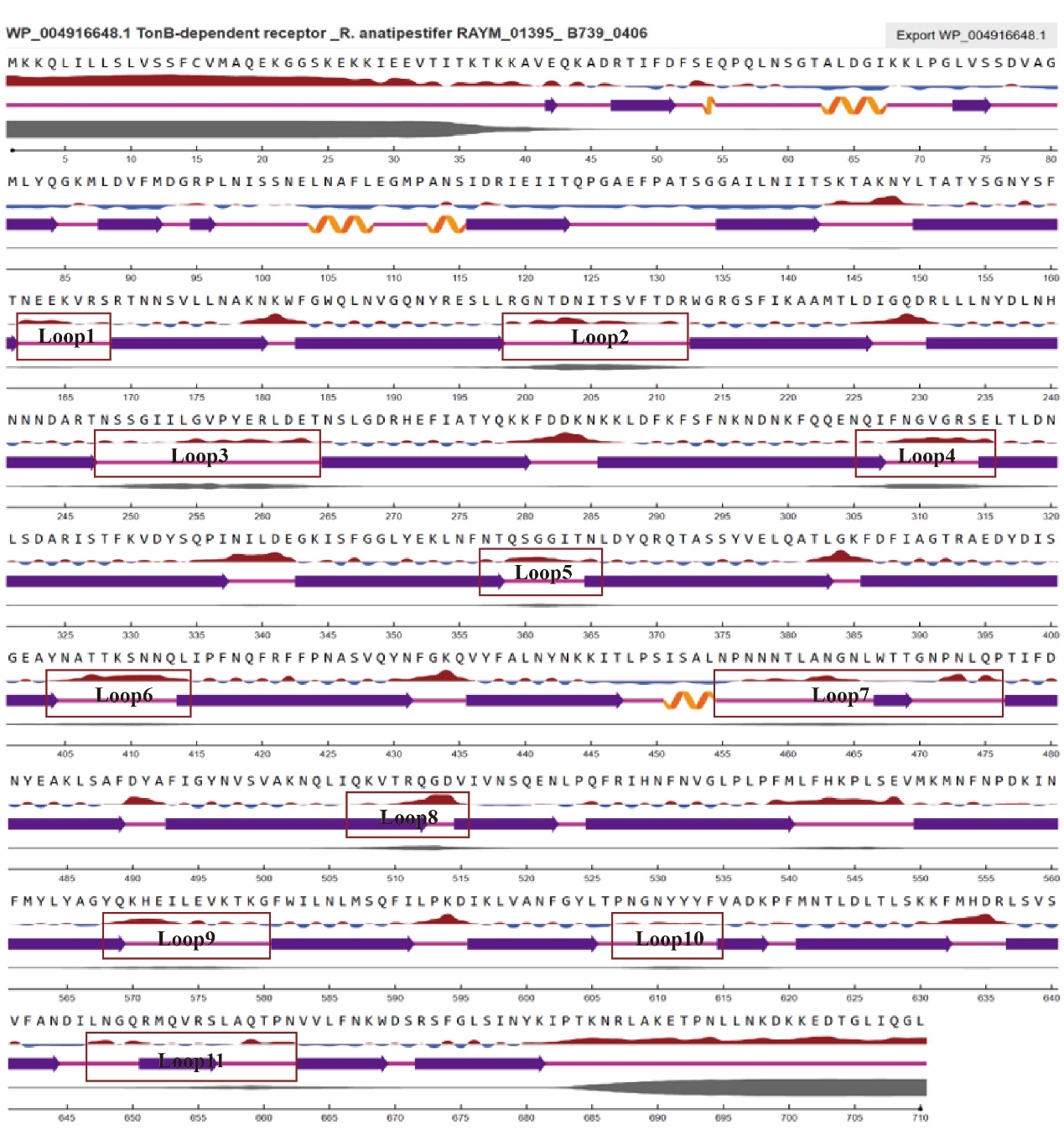


**Supplementary Figure 4.** Secondary structure prediction of OMP76.


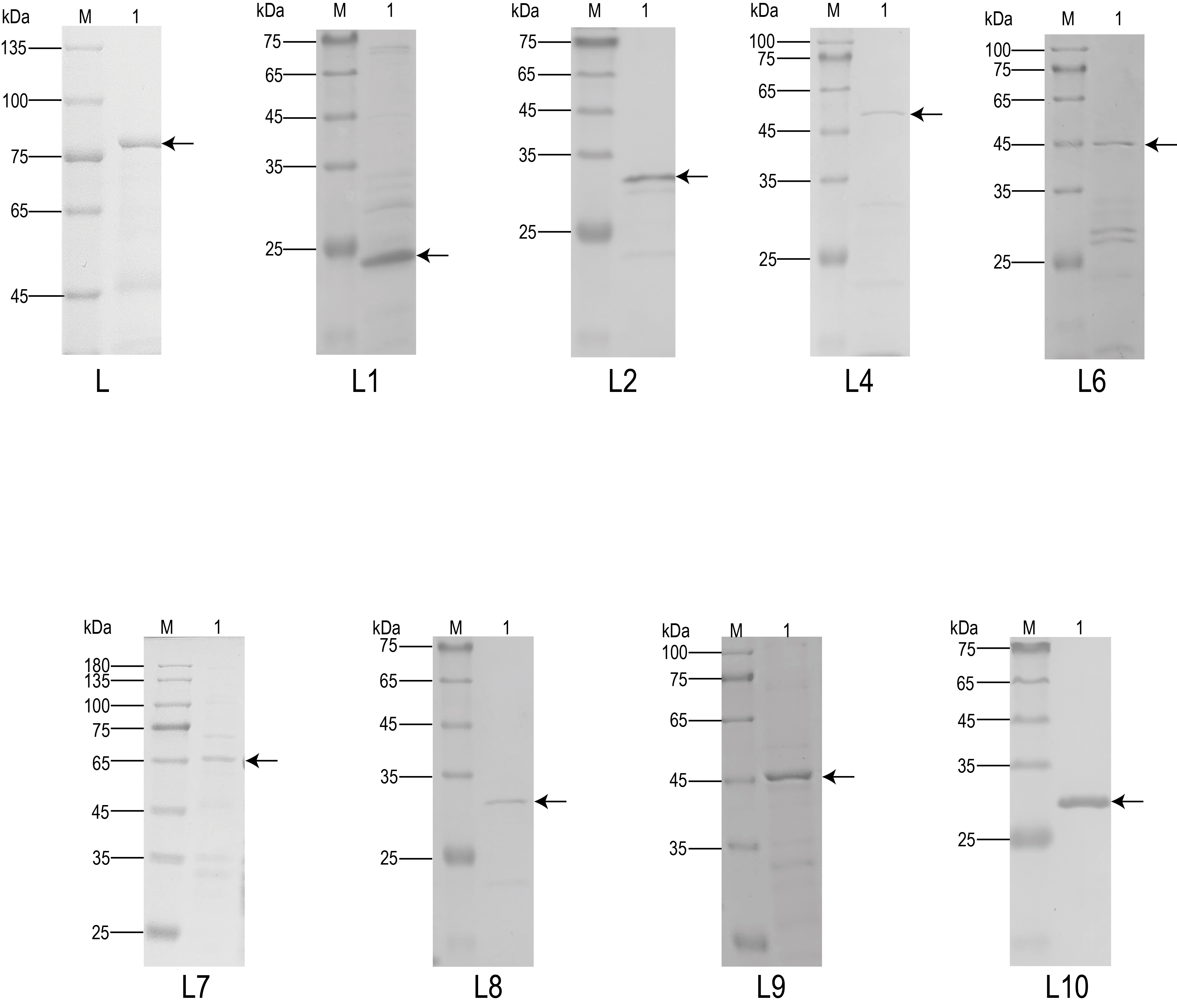


**Supplementary Figure 5**. Purification of truncated fragments of OMP76 assessed by SDS-PAGE. M, molecular weight marker


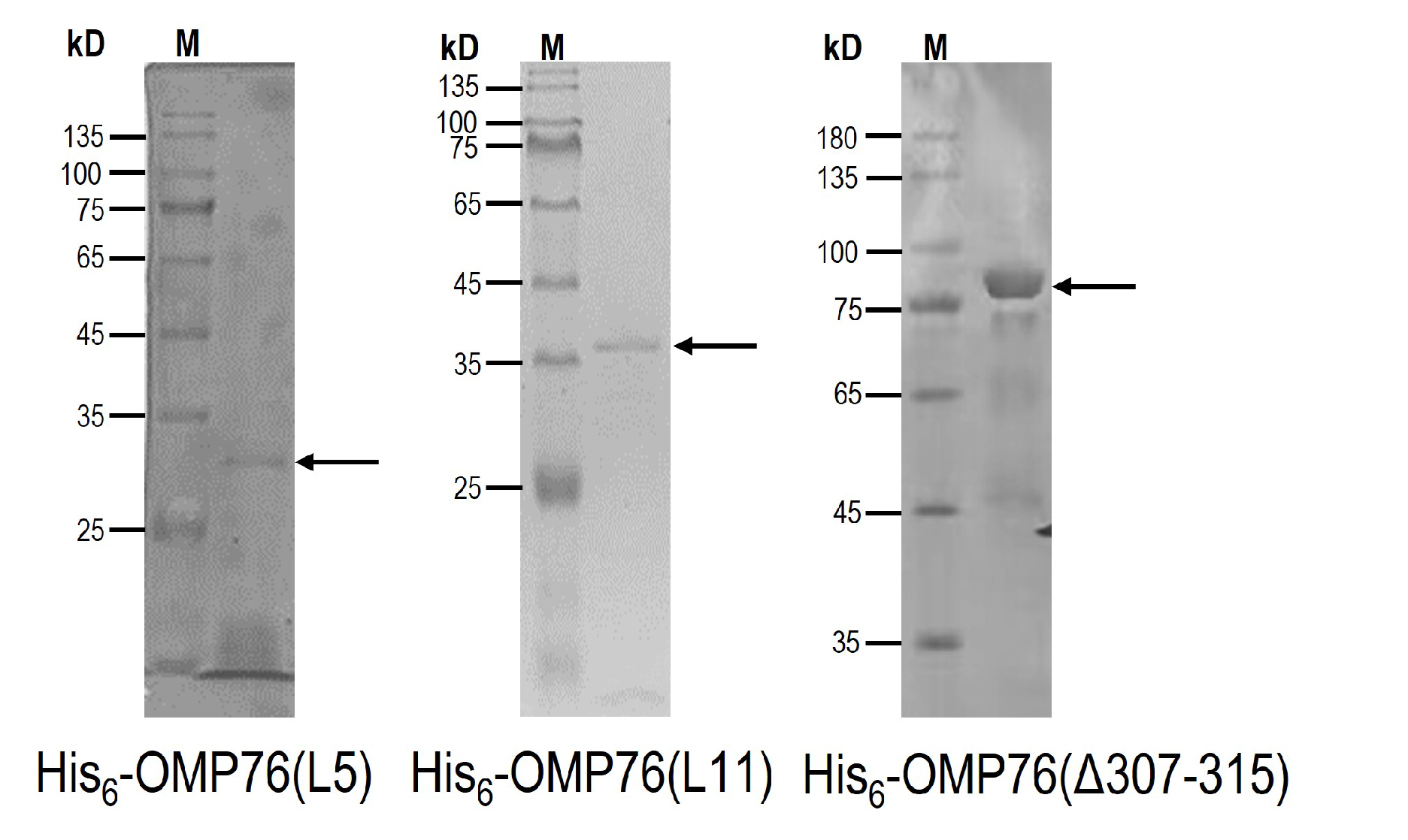


**Supplementary Figure 6.** Purification of truncated fragment L5, L11 and Δ307-315 of OMP76 assessed by SDS-PAGE. M, molecular weight marker.


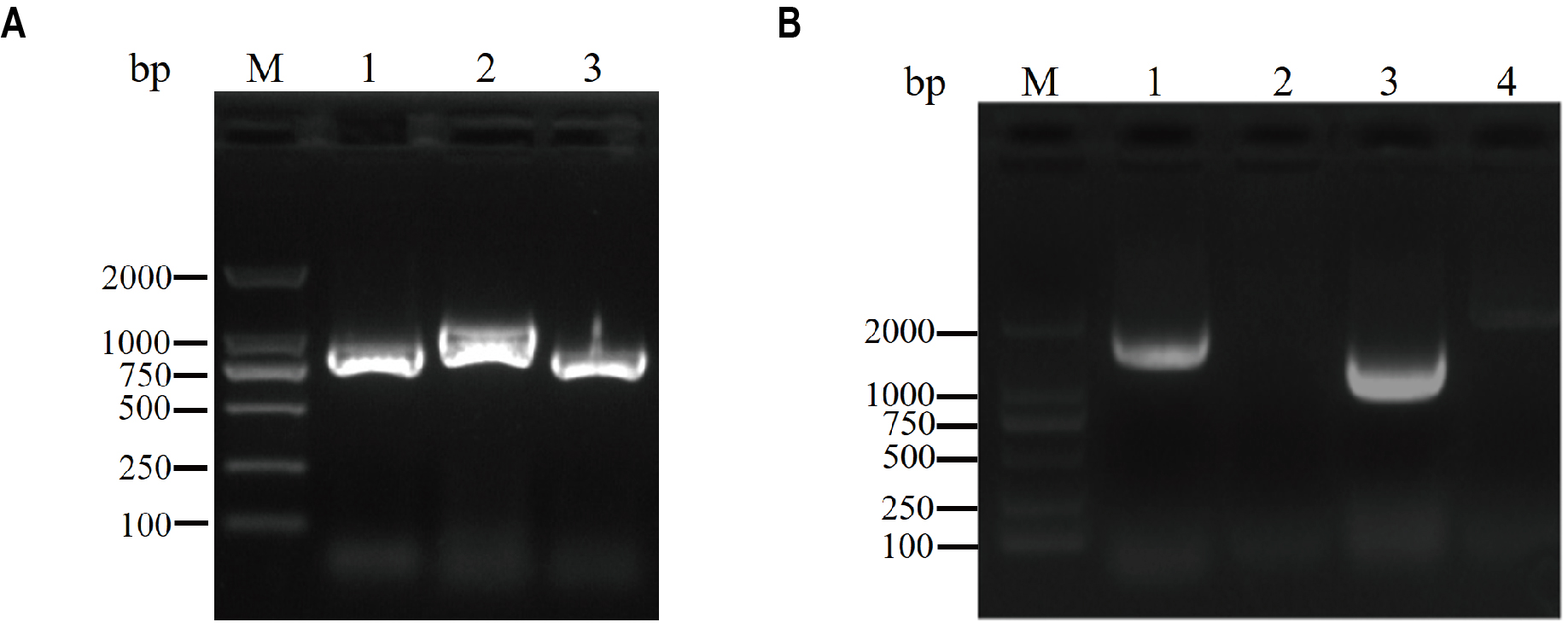


**Supplementary Figure 7.** Construction of *R. anatipestifer* RA-YMΔOMP76. (A) Amplification of the 5’ and 3’ homology arms of *omp76* and the spectinomycin resistance gene. Lanes: M, molecular weight marker; 1, 5’ homology arm; 2, spectinomycin resistance gene; 3, 3’ homology arm. (B) PCR identification of the *omp76* gene deletion strain. Lanes: M, molecular weight marker 1, *R. anatipestifer* 16SRNA; 2, *omp76* gene; 3, spectinomycin resistance gene; 4, negative control.

**Supplementary Table 1.** Strains and plasmids in this study.

| Strains and plasmids | Description | Source |
| --- | --- | --- |
| Strains |  |  |
| RA-YM | *Riemerella anatipestifer* wild-type strain, serotype 1 | Laboratory preservation |
| Δ*OMP76* | *omp76* gene deletion mutant strain, Spec^R^ | This study |
| CΔ*OMP76* | Complemented Δ*omp76* strain, Spec^R^, Erm^R^ | This study |
| *E. coli* DH5α | F- φ80 *lacZΔM15* Δ(*lacZYA-argF*) *U169* *endA1* *recA1* *hsdR17*(rk^-^, mk^+^) *supE44* λ^-^ *thi-1* *gyrA96 relA1 phoA* | This study |
| *E. coli* DH10Bac | F^-^ *mcrA* ∆(*mrr-hsdRMS-mcrBC*) ϕ80*lacZ∆M15 ∆lacX74* *recA1 endA1 araD139* ∆ (*ara, leu*)7697 *galU galK* λ^-^ *rpsL nupG* /pMON14272/pMON7124 | This study |
| *E. coli* BL21 (DE3) | *E. coli* B F^-^ *dcm ompT* *hsdS* (rB‑ mB‑) *gal* [*malB*+] K-12 (λS) | Laboratory preservation |
| *E. coli* X7213 | *thi-1 thr-1 leuB6 glnV44 fhuA21 lacY1 recA1* RP4-2-Tc: Mu λ *pir1 asdA41* *zhf-2*:Tn*10* | Laboratory preservation |
| Plasmids |  | Laboratory preservation |
| pMD18-T | TA cloning vector | TAKARA |
| pRES-JX-Erm | Shuttle vector | Laboratory preservation |
| pRES-JX-Erm-OMP76 | Shuttle vector | This study |
| pET-16b | Expression vector | Laboratory preservation |
| pET-16b-OMP76 | Expression vector | This study |
| pET-22b | Expression vector | Laboratory preservation |
| pET-22b-OMP76 | Expression vector | This study |
| pFastBac | Expression vector | Laboratory preservation |
| pFastBac-Vn | Expression vector | This study |

**Supplementary Table 2.** Primers in this study.

| Primer | Sequence (5’-3’) | Source |
| --- | --- | --- |
| Primers for amplification of dVn and C9 | | |
| C9-F | CGCGGATCCGCCACCATGGGATCATGGAGCCACCCGCAGTTCGAAAAGATGCTTTCGGGAGAAAGCAGAAGCTCTTCAGAAAAAGCTT | This study |
| C9-R | CCGGAATTCTCAACAGGGACGTTTTTCAATGGCTTCTCCTTGGCATGGTCTGCCTCCATCT | This study |
| Vn-F | CGGCTCGAGATGGGATCATGGAGCCACCCTCAGTTCGAAAAGATGGCTGAAGAGTCCTGCGAGGGTCGCTGCGATG | This study |
| Vn-R | CCCAAGCTTTCAGGTGCTCTCCTCCCCGGGCTGCGGGCAGT | This study |
| Primers for construction of Δ*OMP76* and CΔ*OMP76* | | |
| OMP76-CF | TATGCTATAAAATATAACACAATGAAAAAACAATTAATTCTATTGTCTTTAGT | This study |
| OMP76-CR | GTTAGCAGCCGGATCCTCGAGAAGACCTTGGATAAGCCCTGTATC | This study |
| OMP76-F1 | CGGGGTACCATGTATTGTGTGTTAGGATTAAGACTTTTTGAAGTTTTTTT | This study |
| OMP76-F2 | TGAGTTTTCGTTCCACTGTGTGTTATATTTTATAGCATAGTAGGT | This study |
| OMP76-R1 | GCTTACTTTTAAAACTACTGTAGCCAATTTCATTAAGGGAGTTTAAGAAA | This study |
| OMP76-R2 | GGCGAGCTCTAATTACGAGGCTGACTTGATTTTTTAGGAACTCAAAATTA | This study |
| Promoter-OMP76-F | TTGAGAACGATTTAGCATATGAAAGTGGCAAATTTAACTTTAA | This study |
| Promoter-OMP76-R | TAATTGTTTTTTCATTGTGTTATATTTTATAGCATAG | This study |
| Spec-S1 | ATGCTATAAAATATAACACACAGTGGAACGAAAACTCACGTTAAGGGATT | This study |
| Spec-S2 | TCCCTTAATGAAATTGGCTACAGTAGTTTTAAAAGTAAGCACCTGTTATT | This study |
| Primers for *recA*, the internal gene of strain RA-YM | | |
| *RecA*-F | GGAATTCCATATGGCAAAGACTGAAACAACAAG | This study |
| *RecA*-R | CCCAAGCTTGGGCTAGTGGTG TGGTGGTGGTGTTTAGCTTGTAATTTTTCTCTGATTTTTGC | This study |
| Primers for surface expression of pET-16b-OMP76 in *E. coli* BL21 (DE3) | | |
| *OMP76*-SF | CCGCTCGAGATGAAAAAACAATTAATTCTATTGTC | This study |
| *OMP76*-SR | CGCGGATCCAAGACCTTGGATAAGCCCTGTATC | This study |
| Primers for amplification of OMPs of strain RA-YM | | |
| *asnB*-F | CGCGGATCCATGTGTGGCATCTATATAACCAATATACC | This study |
| *asnB*-R | CCGCTCGAGTGAAATTTTCATTTTAAATTCTTCGCACCA | This study |
| *DUF885*-F | CGCGGATCCATGGGAGATACACCTTTTGTTGTAAATACCA | This study |
| *DUF885*-R | CCGCTCGAGCTTTTGGTTTTTTGCCCAAATTTCCATT | This study |
| *FAA1*-F | CGCGGATCCATGAATTTAGCTGCATTTGTAAGTGT | This study |
| *FAA1*-R | CCGCTCGAGAGCATACAACTGCTCTATTAAGTTACTGTAT | This study |
| *OMP76*-F | CGCGGATCCATGCAGGAGAAAGGTGGCTCAAAAGAG | This study |
| *OMP76*-R | CCGCTCGAGAAGACCTTGGATAAGCCCTGTATCTTCT | This study |
| *OMP85*-F | CGCGGATCCATGAAACATATAATCAAACGATATTCAATAC | This study |
| *OMP85*-R | CCGCTCGAGAAAAGGATAGCCTATGGCTATGTTTAGG | This study |
| *PspC*-F | CGCGGATCCATGAACAAAACATTATCAATAGGACTGGCT | This study |
| *PspC*-R | CCGCTCGAGTTTTCTGTTTTTTATACTGATGATAATATCGC | This study |
| *RseP*-F | CGCGGATCCATGGATTTGTTAACCCAAATATTTCA | This study |
| *RseP*-R | CCGCTCGAGTTTAAAAATTTTAAAAATGTCACTTCCAAT | This study |
| *Tadc*-F | CGCGGATCCATGAAAAAAATATTTCAAATTTTAATTTTC | This study |
| *Tadc*-R | CCCAAGCTTGATTAACCACTGCTATTTTCGCTGTTGCTT | This study |
| *Tuf*-F | CGCGGATCCATGGCAAAGGAAACGTTTAATCGTA | This study |
| *Tuf*-R | CCGCTCGAGGTCTAGGATTTCAGTTACCTGACCAGCACCT | This study |
| Primers for amplification of the deletion fragments of *omp76* | | |
| L-F | CCCAAGCTTGAAGAAGTTACCATCACCAAAACC | This study |
| L-R | CCGCTCGAGAAGACCTTGGATAAGCCCTGTATC | This study |
| L1-F | CCCAAGCTTGAAGAAGTTACCATCACCAAAACC | This study |
| L1-R | CCGCTCGAGTCTAAGTAGAGATTCTCTGTAATTTTG | This study |
| L2-F | CCCAAGCTTGAAGAAGTTACCATCACCAAAACC | This study |
| L2-R | CCGCTCGAGTATTTGATTTTCCTGCTGGAATTTG | This study |
| L3-F | CCCAAGCTTGAAGAAGTTACCATCACCAAAACC | This study |
| L3-R | CCGCTCGAGGTCTCCCTGACGAGTTACTTTC | This study |
| L4-F | CCCAAGCTTGAAGAAGTTACCATCACCAAAACC | This study |
| L4-R | CCGCTCGAGTTGGTAGCCCGCATAGAGGTACA | This study |
| L5-F | CCCAAGCTTTCTTTTACCAATGAAGAAAAAGTAC | This study |
| L5-R | CCGCTCGAGTTGGGTATTAAAATTCAGCTTCTCGTAC | This study |
| L6-F | CCCAAGCTTTCTTTTACCAATGAAGAAAAAGTAC | This study |
| L6-R | CCGCTCGAGTTGGTAGCCCGCATAGAGGTACA | This study |
| L7-F | CCCAAGCTTTCTTTTACCAATGAAGAAAAAGTAC | This study |
| L7-R | CCGCTCGAGAAGACCTTGGATAAGCCCTGTATC | This study |
| L8-F | CCCAAGCTTTTGACATTAGACAATCTATCAG | This study |
| L8-R | CCGCTCGAGTTGGTAGCCCGCATAGAGGTACA | This study |
| L9-F | CCCAAGCTTTTGACATTAGACAATCTATCAG | This study |
| L9-R | CCGCTCGAGAAGACCTTGGATAAGCCCTGTATC | This study |
| L10-F | CCCAAGCTTGACGTGATTGTTAATAGTCAAG | This study |
| L10-R | CCGCTCGAGAAGACCTTGGATAAGCCCTGTATC | This study |
| L11-F | CCCAAGCTTTCTTTTACCAATGAAGAAAAAGTAC | This study |
| L11-R | CCGCTCGAGTACATAAGACGAGGCGGTTTGC | This study |
| Δ307-315-F1 | CCCAAGCTTGAAGAAGTTACCATCACCAAAACC | This study |
| Δ307-315-R1 | TTGATTTTCCTGCTGGAATTTGTTATCA | This study |
| Δ307-315-F2 | AAATTCCAGCAGGAAAATCAATTGACATTAGACAATCTATCAG | This study |
| Δ307-315-R2 | CCGCTCGAGAAGACCTTGGATAAGCCCTGTATC | This study |

**Supplementary Table** **3.** Outer membrane proteins most likely to interact with dVn.

| **Protein** | **Accession Number** | **Sum PEP Score** | **Percent Cover** | **Peptides** | **Unique Peptides** | **MW**  **(kD)** |
| --- | --- | --- | --- | --- | --- | --- |
| SecA | WP_004916348.1 | 52.17 | 19 | 20 | 20 | 116.9 |
| TonB-dependent receptor | WP_004916648.1 | 25.31 | 12 | 8 | 8 | 80.4 |
| RIP metalloprotease RseP | WP_004916196.1 | 18.34 | 12 | 5 | 5 | 50 |
| Elongation factor Tu | WP_004916760.1 | 17.84 | 14 | 5 | 5 | 43.2 |
| Long-chain fatty acid CoA ligase | WP_004918735.1 | 9.73 | 11 | 4 | 4 | 65.7 |
| Hypothetical protein | WP_004917060.1 | 7.78 | 5 | 4 | 4 | 89 |
| Asparagine synthase | WP_004918287.1 | 8.15 | 5 | 3 | 3 | 70.3 |
| DUF885 domain-containing protein | WP_004917066.1 | 9.90 | 5 | 3 | 3 | 69 |
| T9SS C-terminal target domain-containing protein | WP_004919973.1 | 5.52 | 4 | 3 | 3 | 83.1 |
| PspC | WP_004919472.1 | 5.41 | 5 | 3 | 3 | 63 |

**Supplementary Materials and Methods**

**Recombinant baculovirus construction and protein purification**

Total RNA was extracted from Cherry Valley duck liver and used for cDNA production by reverse transcription PCR using Evo M-MLV RT Mix Kit (Accurate Biology AG, Changsha, China). The duck vitronectin gene (1311 bp) deleted of the signal peptide sequence and engineered with a Strep tag II for protein purification and the duck complement factor C9 gene (1716 bp) were amplified by PCR using duck liver cDNA as template and primers Vn-F/Vn-R and C9-F/C9-R, respectively (Supplementary Table 2). The genes were cloned into the pFastBac1 plasmid and transformed into *E. coli* DH10Bac. Recombinant baculoviruses Bacmid-Vn and Bacmid-C9 packaged in this strain were extracted using the Endo-free BAC/PAC DNA Maxi Kit (Omega Bio-Tek, Norcross, USA) and selected using CellfectinTM II (Gibco) following transfection into Sf9 insect cells. Viral titers were raised by infecting the Sf9 cells four times successively with the recombinant baculovirus to achieve a viral titer of 1×109 pfu/ml to facilitate bulk purification of dVn proteins. The infected cells were collected by centrifugation and resuspended in lysate buffer (100 mM Tris-HCl, 150 mM NaCl, 1 mM EDTA, pH 8.0). Impurities were removed by centrifugation at 10000×g for 30 min after high-pressure disruption and the supernatant was passed through a 0.22 μm Millex®-GP filter (Merck Millipore, Darmstadt, Germany). The sample was loaded onto an equilibrated Strep Trap HP column (Cytiva Sweden AB, Uppsala, Sweden) and the protein was eluted with elution buffer (100 mM Tris-HCl, 150 mM NaCl, 1 mM EDTA, 2.5 mM desthiobiotin, pH 8.0). Desthiobiotin was removed using a PD-10 desalting column (Cytiva Sweden AB). SDS-PAGE followed by western blotting with a mouse anti Strep II-Tag mAb (ABclonal, Wuhan, China) were used to confirm the purified protein.

**Cloning, expression and purification of His_6_-OMPs, His_6_-RecA and His_6_-OMP76 truncated fragments**

The *recA* sequence of the *R. anatipestifer* internal reference gene for western blotting and the sequences of genes for candidate proteins for interactions based on LC-MS/MS results were amplified by PCR and cloned into the pET-32a plasmid using the RA-YM strain genome as a template (Supplementary Table 2 for primer sequences). The recombinant pET-32a plasmids were transformed into *E. coli* BL21 (DE3) and grown to OD_600_ = 0.6-0.8. Expression of the cloned genes was induced with IPTG (1 mM) at 27^o^C for 6 h. The cells were collected and resuspended in binding buffer (20 mM Na_3_PO_4_, 500 mM NaCl, 30 mM imidazole, pH 7.4). Cells were subjected to high-pressure (1000 bar) disruption three times (JNBIO, Guangzhou, China) and the lysate was centrifuged to remove insoluble impurities. His_6_-OMPs and His_6_-RecA in the supernatant were purified using His-trap HP (Cytiva Sweden AB). After sample loading, the column was washed with binding buffer, and the proteins of interest were eluted with elution buffer (20 mM Na_3_PO_4_, 500 mM NaCl, 30-500 mM imidazole, pH 7.4). Similarly, expression and purification of the OMP76 truncated and knockout fragments produced from pET-22b or pET-32a were performed according to the procedure described above. Vector pET-16b was used for surface expression of OMP76 in *E. coli* BL21 (DE3).
